# Development of a Non-Invasive Method, Multiplex Methylation Specific PCR (MMSP), for Early Diagnosis of Nasopharyngeal Carcinoma

**DOI:** 10.1371/journal.pone.0045908

**Published:** 2012-11-05

**Authors:** Zhe Zhang, Di Sun, Susanna Hilda Hutajulu, Imran Nawaz, Do Nguyen Van, Guangwu Huang, Sofia M. Haryana, Jaap M. Middeldorp, Ingemar Ernberg, Li-Fu Hu

**Affiliations:** 1 Department of Microbiology, Tumor and Cell Biology (MTC), Karolinska Institutet, Stockholm, Sweden; 2 Department of Otolaryngology-Head and Neck Surgery, First Affiliated Hospital of Guangxi Medical University, Nanning, Guangxi, China; 3 Molecular Biology, Faculty of Medicine, Gadjah Mada University, Yogyakarta, Indonesia; 4 Department of Pathology, VU Medical Center, Amsterdam, The Netherlands; 5 Department of Immunology and Pathophysiology, Hanoi Medical University (HMU), Dongda, Hanoi, Vietnam; Institute of Cancerology Gustave Roussy, France

## Abstract

Increasing evidence demonstrated that inactivation of tumor suppressor genes (TSGs) by aberrant promoter methylation is an early event during carcinogenesis. Aiming at developing early diagnostic or prognostic tools for various tumors, we took an EBV-associated tumor, nasopharyngeal carcinoma (NPC), as a model and developed a powerful assay based on “multiplex methylation specific-PCR (MMSP)”. The MMSP assay was designed to detect tumor-specific methylation status of several NPC-related genes and was capable of acquiring multiplex information simultaneously through a single PCR reaction with the tiny tumor DNA derived from the direct body fluid close to the primary tumor. In this study, we collected paired nasopharyngeal (NP) swabs and NPC biopsies from 49 NPC patients and twenty noncancerous controls. A panel of markers including two EBV, and two cellular TSG markers were applied in this NPC-specific-MMSP assay. We optimized the working condition of MMSP so that it provides information equal to that from the corresponding separate PCRs. The results showed that MMSP patterns of NPC swab were largely consistent with those of corresponding biopsies and significantly distinguished themselves from those of 20 noncancerous volunteers. Among the 69 samples (49 NPCs and 20 normal controls), the sensitivity of detecting NPC from NP swabs is 98%. The specificity is as high as 100%. In conclusion, being characterized by its noninvasiveness, high reproducibility and informativeness, MMSP assay is a reliable and potential diagnostic tool for NPC. It paves the way for the development of population screening and early diagnosis approaches for various tumor types.

## Introduction

Nasopharyngeal carcinoma (NPC) is the second most common cancer in southern China and constitutes the main menace in this area. Patients in stage I and II disease have a significantly longer overall survival compared with those in stage III and IV. The 5-year survival rate for stage III or IV patients was only 54.5%, while it could be as high as 95% for stage I or II patients [1]. So, early diagnosis for NPC is essential in achieving a satisfactory therapy effect. Unfortunately, because of the non-specific local symptoms and the un-convenience of a fully clinical examination of the nasopharynx, the majority of NPC patients are only diagnosed when the tumor has reached an advanced stage [2]. Approximately 70% of newly-diagnosed NPC patients presented as advanced diseases with a poor prognosis [2], [3]. Thus, to find biomarkers for detecting early stage NPC and monitoring residual or recurrent tumor would help greatly in elevating survival probability and the choice of subsequent therapy.

As a 100% Epstein-Barr virus (EBV)-related tumor, the attempts for early diagnosis of NPC have been largely associated with the characteristics of EBV infection. Elevated serum titers of IgA antibodies to viral capsid antigen (VCA) and early antigen (EA) have been the most commonly used markers for screening and monitoring of the disease [4]. However, the sensitivity and specificity of this serological method are known to be low [5], [6]. Measurement of EBV load by real-time PCR claimed a high sensitivity based on laboratory studies, but its cut-off is hard to define as a single marker for diagnosis [7].

Epigenetic silencing of tumor suppressor genes (TSGs) by promoter methylation is an early event in the multi-step process of carcinogenesis. Aberrant methylation of *p16* gene and *O^6^-MGMT* gene can already be detected from the patients with squamous cell lung carcinoma three years prior to clinical diagnosis [8]. Frequent aberrantly methylated TSGs in tumors have been used as molecular markers for the detection of malignant cells from various clinical materials. It provides possibilities of both cancer early detection and dynamic monitoring of cancer patients after treatment [9]. Aberrant methylation on TSG promoters is rather prevalent and tumor-specific among NPCs, which suggests its potential as an assay for NPC diagnosis. Several technical approaches are now available for analyzing DNA methylation status, such as Southern blot analysis, bisulfite genomic sequencing, methylation specific PCR (MSP), and methylation-sensitive restriction enzyme digestion. Although considerable amounts of DNA are incorporated in these methods, the number of genes investigated in a single reaction is still limited. Recently, some approaches that allow high-throughput analyses of multiple CpG islands were developed, including methylation specific oligonucleotide microarray, restriction landmark genomic scanning, and differential methylation hybridization [6], [10], [11], [12], [13]. However, these techniques require sophisticated equipments for bioinformatic analyses, which limits its prevalence in the clinic.

In order to develop a method which is both informative and starting-material-saving, we designed an assay named multiplex methylation PCR (MMSP), by doing which we were aiming at getting methylation-based tumor-suggestive information from an optimal panel of markers including TSGs and EBV genes for the early detection of NPC. The MMSP assay was designed to provide the following information. 1) EBV infection. It helps to distinguish EBV-related tumors from EBV-unrelated tumor or normal cells. In this MMSP assay, a fragment of *EBNA1* gene was amplified as an evidence of EBV infection of tumor cell. 2) The expression status of EBV-encoded oncogenic latent membrane protein (LMP1). LMP1 is the main EBV transforming protein in NPC. It could be unequivocally detected in 65% of NPC patients [14]. It has been shown to be tightly correlated with its promoter methylation [15]. NPC biopsies with unmethylated *LMP1* promoter always express LMP1 protein. 3) The methylation status of two cellular candidate TSGs: Ras association domain family protein 1, isoform A (*RASSF1A*) and Death-associated protein kinase (*DAPK*) genes. These two genes have been shown to be heavily methylated in 84% and 80% in NPC biopsies respectively, but with few exceptions not in normal NP epithelia [16], [17], [18].

The aim of the present study is to evaluate the capability of MMSP assay of detecting multiplex information and feasibility for diagnosis of NPC using tumor cells from NP swab as material. The MMSP assay demonstrated here is capable of simultaneously detecting methylation status of multiple genes, which are key regulators of diverse fundamental pathways. The informativeness and amenability of MMSP makes it suitable for clinical use.

## Materials and Methods

### Cell lines, NPC tumor specimens, normal nasopharyngeal epithelia and paired NP swabs

EBV-positive latency III Burkitt lymphoma cell line, Namalwa [19], has been shown to contain two copies of the EBV genome in each single cell and expresses all the six nuclear protein EBNA1-6 and three latent membrane proteins LMP1, LMP2A and 2B. Furthermore, *RASSF1A* and *DAPK* were demonstrated to be methylated in Namalwa cells according to our data. It was used as a positive control to test the robustness of MMSP technique in this study. It was cultured in RPMI medium at 37°C with 5% CO_2_. Human NPC cell lines CNE1, CNE2 (EBV negative, from Cancer Center, gift from Jianjing Chen, Sun Yat-sen University, and established by Zeng Yi, Chinese Academy of Preventive Medicine, China) and TW03 (EBV negative, established by and a gift from CT Lin, National Taiwan University Hospital) [20] were cultured in IMEM (Gibco USA) containing 10% fetal calf serum (FCS). The immortalized nasopharyngeal epithelial cell line NP69 (EBV negative, established by and a gift from SW Tsao, the University of Hong Kong, China) [20] was cultured in keratinocyte serum-free medium (Invitrogen, Carlsbad, CA) supplemented with 25 µg/ml bovine pituitary extract, and 0.2 ng/ml recombinant epidermal growth factor, as suggested by the manufacturer. C666-1 is an NPC cell line with weak EBV positive (gift and established by Dolly Huang, Chinese University of Hong Kong) and was kept in IMDM with 10% FCS. C15 is an African-origin, EBV-positive and LMP1-expressing NPC xenograft [21] (gift from Dr. P. Busson, Institute Gustave Roussy, Paris). Forty-nine paired biopsies and swab from pathology-verified NPC patients and twenty normal volunteers were obtained and informed at First Affiliated Hospital of Guangxi Medical University (approved by the Ethical Review Committee of First Affiliated Hospital of Guangxi Medical University and Ethics Committee, Karolinska Institute, Stockholm, Sweden, Ref.no:00-312). Patients gave their informed consent verbally, which we recorded in the patient's file (history record). The paired NP swabs were collected from each subject by inserting cotton-tip sticks blindly from both the right and left nose to the nasopharyngeal wall. In brief, the patient's nasal cavity was sprayed with a 1% cocaine solution for superficial anesthesia. Cotton sticks were then inserted into the nasal cavity and moved until touch the nasopharyngeal wall. The cotton was swabbed against the posterior and lateral nasopharyngeal walls for several times. Then the cotton stick was withdrawn, and the cells were collected by dipping the cotton tip into 2 ml of saline and vortex for 15 second. After taking out the cotton tips, cell pellets were collected by centrifugation and were stored at −80°C until use.

### DNA extraction and bisulfite modification

Homogenized biopsies and swab cell pellets were treated with TE buffer containing 0.5% SDS and 50 µg/ml proteinase K (Invitrogen, Carlsbad, CA) for 3 hours at 56°C. High molecular weight genomic DNA was obtained by conventional phenol/chloroform and ethanol extraction. The bisulfite modification procedure was slightly modified according to the protocol of Alexander Olek et al [22]. In brief, 500 ng of genomic DNA in 9 µl volume was denatured by heating at 95°C for 5 minutes and immediately transferred to ice for 5 minutes. One µl of 3 M NaOH was then added and incubated for 15 min at 37°C. It was then neutralized with 10 µl of H_2_O and two volumes (40 µl) of 2% low melting point agarose was added into it. Thirty µl agarose/DNA mixtures were pipetted into two tubes containing 100 µl of chilled mineral oil to form agarose bead. Each bead was placed in an individual tube to which 200 µl of 5 M bisulphate solution (2.5 M sodium metabisulphite, Sigma; 100 mM hydroquinone, Sigma; pH5.0) was then added. The reaction mixture was incubated in darkness for 16 h at 50°C. Treatment was stopped by equilibration against 1 ml of TE buffer followed by desulphonation in 500 µl of 0.2 M NaOH. Finally, the beads were washed with 1 ml of H2O, and then used directly in PCRs.

The average amount of DNA equals 10^6^–10^7^ cell equivalents/brush and is quite reproducible per individual brush according to the study by Stevens et al [23]. In our work, we used cotton swabs, due to lower cost. The DNA recovery from these is slightly lower than that from brush, but the amount (5–15 µg DNA) and quality of DNA was validated to be good for PCR based experiments. The same protocol was used for small amounts of DNA with salmon DNA as carrier.

### Multiplex Methylation Specific PCR (MMSP)

A cocktail of gene-specific primers was used to co-amplify *EBNA1*, unmethylated *LMP1* (U-*LMP1*), and methylated *RASSF1A* (M-*RASSF1A*) and *DAPK* (M-*DAPK*). A house-keeping gene *β*-*ACTIN* was also included in the MMSP assay to monitor the quality and quantity of input template DNA and the efficiency of bisulfite conversion. Corresponding single MSPs were also performed to confirm the accurateness and density of amplicons. The sequences of PCR primer sets specific for methylated and unmethylated alleles of expected PCR product are summarized in table 1. For PCR reaction, 2 µl of bisulfite-modified DNA (16.6 ng) was added in a final volume of 25 µl of PCR mixture containing 2× PCR buffer, 3.5 mM MgCl_2_, 100 pmol deoxynucleotide triphosphates, primers (100 pmol each per reaction) and one unit of AmpiTaq Gold (Applied Biosystems, Branchburg, NJ). PCR amplification was performed at 95°C for 10 min, then followed by 34 cycles at 94°C for 30 s, 55°C for 45 s, and 72°C for 90 s. PCR products were analyzed by 3% agarose gel electrophoresis stained with ethidium bromide.

**Table 1:** Summary of the primers used in the present study.

Primers	Sequences	Product size
*EBNA1*-Forward	5′AGGGTTAAGATATAGAGATGGTGTT 3′	129 bp
*EBNA1*-Reverse	5′ TACTCCTACCCCTCCTACTCCTAC 3′	
U-*LMP1*-Forward	5′ TGGTTATGTTAGAGTAATGTG 3′	149 bp
U-*LMP1*-Reverse	5′ TTTCTACTTCCCCTTTCTATC 3′	
M*-RASSF1A*- Forward	5′GTTTTGCGAGAGCGCG 3′	169 bp
M*-RASSF1A*- Reverse	5′ GGTAACAAACGCGAACG3′	
*ß-ACTIN* -Forward	5′ AAGTTAAGTTTTGTTTTTATTTTT3′	184 bp
*ß-ACTIN* -Reverse	5′ CAATAATCTCCTTCTACATCCTATC3′	
M*-DAPK*-Forward	5′ CGGTAGGGTTTGGGGTCG 3′	227 bp
M*-DAPK*-Reverse	5′ AAACCTCCCAACTTCGATCG 3′	

## Results

### The feasibility of MMSP

**Figure 1:**
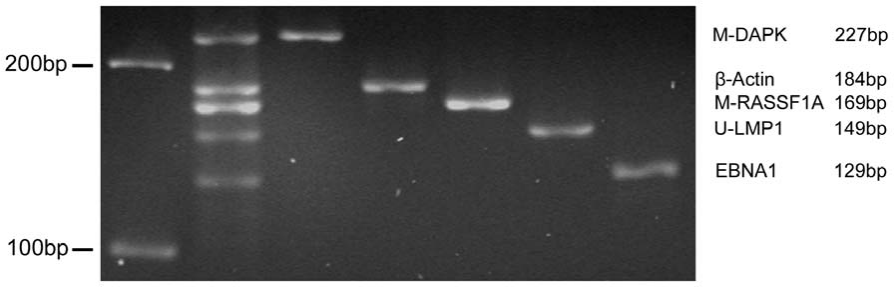
Comparison of MMSP and corresponding single MSP assays. Same amount of bisulfite converted genomic DNA of Namalwa cells were used as template for both multiplex and single MSPs. All the markers demonstrated highly specific bands in this MMSP assay. There was an excellent concordance with the relative density of the five bands including *β-ACTIN* as internal control between MMSP and single MSPs.

In order to test the feasibility of MMSP to achieve the same readout as those from single MSPs, we used Namalwa cell line as a testing material. All markers in the MMSP system were supposed to show positive bands for bisulfite-treated Namalwa DNA. We compared the specificity and efficiency of MMSP assay using the corresponding five single MSPs as control. As shown in Figure 1, all the markers demonstrated highly specific bands in this MMSP assay. And there was an excellent concordance with the relative density of the five bands between MMSP and separate PCR reactions. These results suggest that MMSP assay is tenable both in principle and in reality.

### Detection of MMSP pattern in NPC cell lines

**Figure 2:**
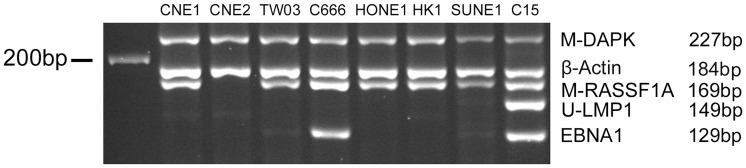
Multiplex Methylation-specific PCR analysis in NPC cell lines and xenograft. All of the cell lines and xenograft showed aberrant methylation on at least one of the two TSGs included in the MMSP assay. The presence of EBV can be only detected in the C666-1 cell line and the EBV-positive xenograft C15. Unmethylated-*LMP1* can be only detected in LMP1 expressing xenograft, C15. The other cell lines demonstrated negative bands for *EBNA1* and *LMP1* markers due to loss of EBV during cell passage.

A series of NPC cell lines and xenografts were included in this study to further validate the reproducibility of MMSP assay. Among these seven NPC cell lines/xenografts, C666-1 and C15 are the only EBV-positive ones. C15 is also an LMP1 non-expressing cell line with heavily-methylated *LMP1* promoters as confirmed by Western blot and bisulfite sequencing. The other five cell lines: CNE1, CNE2, HONE1, SUNE1 and HK1, are all EBV negative NPC cell lines [20], [21]. As shown in Figure 2, the hallmark for the presence of EBV in the MMSP assay, *EBNA1*, could be detected in the C666-1 cell line and the EBV-positive xenograft C15. Unmethylated-*LMP1* could only be detected in LMP1 expressing xenograft, C15. All the rest of cell lines, which were EBV-negative, did not demonstrate bands for *EBNA1* and *LMP1* markers but they all showed aberrant methylation on at least one of the two TSGs included in the MMSP assay. The pattern for these five markers in separate MSP reactions was invariably reproduced in all the samples tested by the MMSP system, which further validate the robustness of MMSP assay.

### Determining the sensitivity of MMSP assay

**Figure 3:**
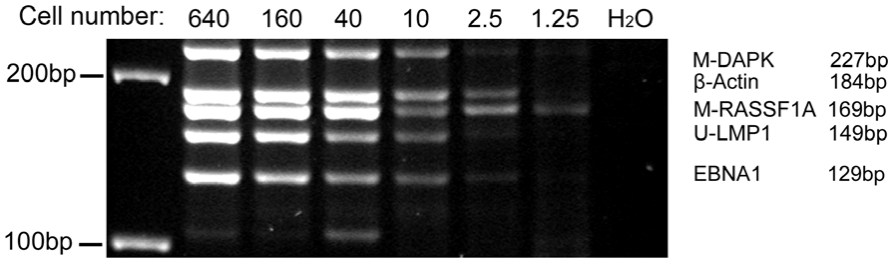
Evaluation of the sensitivity of the MMSP assay. A serial dilution of genomic DNA equal to specific number of Namalwa cells were bisulfite treated and used as templates for MMSP assays. Multiple markers can be co-amplified by a single reaction of MMSP from as few as 10 Namalwa cells.

To evaluate the sensitivity of the MMSP assay, a serial dilution of DNA from certain number of Namalwa cells was performed. The serial diluted DNA was equal to DNA extracted from 640 cells, 160 cells, 40 cells, 10 cells, 2.5 cells and 1.25 cells was then bisulfite modified respectively and used as templates for MMSP assays. As shown in Figure 3, the known MMSP pattern with five markers could be clearly and faithfully exhibited with one single reaction of MMSP from as few as 10 Namalwa cells. Using the conversion estimate of 5 pg/copy of genome DNA, our data demonstrated an extremely high sensitivity of detecting multiple biomarkers from a minimum starting material of 50 pg tumor DNA.

### Detection of MMSP patterns in NP biopsies and corresponding swabs

**Figure 4:**
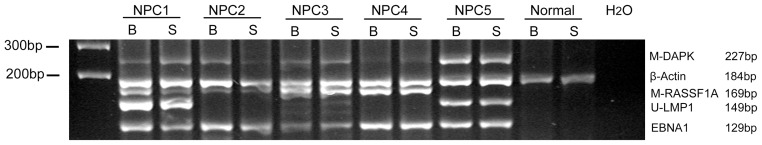
MMSP analysis of paired biopsies and swabs from NPC patients and noncancerous controls. Matched biopsies and swab samples from five NPC patients (NPC 1, 2, 3, 4 and 5) and one noncancerous control were showed as examples. Water was used as a blank control. B: biopsy; S: swab.

Forty-nine NPC biopsies and their corresponding nasopharynx swabs were included in this study to examine the reliability of NP swabs as the origin of material for MMSP assay. Twenty paired NP biopsies and swabs from noncancerous volunteers were also included as normal control. The results were summarized in Table 2. Representative MMSP results are shown in Figure 4. All swab samples from NPC patients and noncancerous controls showed easily detectable bands of the *β-ACTIN* gene in our MMSP analysis, indicating sufficient yields of genomic DNA and successful bisulfite conversion. Presence of EBV latent infection in the biopsy and swab samples was confirmed by amplifying EBV-encoded *EBNA1* gene in the MMSP reaction. EBV DNA was detected in 100% (49 of 49) of NPC primary tumors and 98%(48 of 49) of their paired swab samples, but in none of the noncancerous controls. Sixty-three percent of NPC primary tumors and 55% of their corresponding swab samples showed unmethylated-*LMP1* bands in the MMSP assay, suggesting LMP1 is expressed in these samples. The ratio of U-*LMP1* in NPC samples is quite close to the reported ratio of LMP1 expression by western blot (65%) in NPC. The frequencies of promoter hypermethylation of *RASSF1A* and *DAPK* were 79.6% (39 of 49) and 67.3% (33 of 49) in NPC biopsy samples, while methylated in 57.2% (29 of 49) and 55.1% (27 of 49) respectively in the corresponding swabs.

**Table 2:** Summary of MMSP pattern in NPC and noncancerous controls.

	*EBNA1*		U-*LMP1*		M-*RASSF1A*		M-*DAPK*		*EBNA1* +Any Other	
	Biopsy	Swab	Biopsy	Swab	Biopsy	Swab	Biopsy	Swab	Biopsy	Swab
NPC	49/49	48/49	31/49	27/49	39/49	29/49	33/49	27/49	49/49	48/49
coincidence	98%		87.1%		74.4%		81.8%		98%	
Normal	0/20	0/20	0/20	0/20	0/20	0/20	0/20	0/20	0/20	0/20
coincidence	100%		100%		100%		100%		100%	

In swab samples of NPC patients, 18.4% (9 of 49) exhibited tumor suggestive signal (positive PCR band) from all the four markers, 34.7% (17 of 49) from three of the four markers, 42.9%(21 of 49) from two markers and 2.0% (1 of 49) from one marker only. The MMSP pattern of 42.9% NP swabs (21 of 49) matched exactly with those of corresponding biopsies. If we regard the presence of EBV DNA with tumor suggestive information from any one of the other three markers as a diagnostic criterion for NPC, we reached a sensitivity of 98% in detecting NPC, which covers all the stages of NPC patients. No EBV or methylated markers were ever found in swabs of the noncancerous controls, suggesting a specificity of 100% (zero false-positive).

## Discussion

Gaining sufficient and high-quality tumor DNA is critical for the early diagnosis of cancer. A variety of body fluids and sampling methods have been attempted for the non-invasive diagnosis of NPC, such as serum or plasma from peripheral circulation, buffy coat, and NP swab. The quantity and integrity of cell-free DNA fragments from peripheral circulation specimens such as serum and plasma are usually compromised. Thus, the detection rates of methylated DNA from serum or plasma were proved to be very low [24]. Buffy coat yields DNA of higher quantity and quality; however the DNA is mainly from peripheral lymphocyte instead of from elapsed tumor cells. Among the noninvasive sampling methods, NP swab achieved the highest sensitivity and specificity since it gathers tumor cells by contacting the primary tumor directly [24]. NP swab has also other advantages such as ease of application and swiftness. These attributes together make NP swab extremely amenable for the diagnostic purpose.

A study by Esteller *et al* has shown that a panel of three to four markers could define an abnormality in 70–90% of each cancer type through detecting their aberrant methylation [25]. Further study showed that the EBV methylom present in cancer cells infected with EBV are significantly different as compared to that in human cells corresponding to benign diseases or in non-tumorigenic human B-cell-derived lymphoblastoid cell lines. Thus, epigenetic signatures from both human cellular genes and Epstein-Barr viral genes will be an ideal combination to discriminate NPC from malignancies of other origins [26]. In the present assay, four tumor-related markers and one quality control marker were included. *EBNA1*, a nuclear antigen encoded by EBV, was used as a marker for the evidence of EBV infection. The primers for *EBNA1* were designed to amplify bisulfite-converted EBV genome, but do not distinguish methylated and unmethylated CpGs. The intensity of this marker for EBV presence has the potential of being semi-quantified for EBV load using Namalwa cell as reference. Few malignancies express the EBV-encoded onco-protein, LMP1. The *LMP1* primers used in the assay specially amplified bisulfite-converted unmethylated alleles. Knowledge of LMP1 expression status in a specific NPC will help to further increase the specificity of MMSP and potentially provide information about the prognosis of NPC. In addition, the primers for two cellular genes were designed to amplify their methylated alleles only. *RASSF1A* is a strong candidate TSG for NPC. The RASSF1A protein could interact with DNA repair system and also induce cell cycle arrest. It has been shown to be frequently methylated in NPC biopsies and the aberrant methylation is tightly correlated with loss of expression of RASSF1A in NPC [27]. DAPK is a positive mediator of the programmed cell death induced by gamma-interferon. *DAPK* aberrant methylation is one of the most frequent epigenetic inactivation events detected in NPC. It has also been demonstrated to be associated with aggressive and metastatic phenotype in many human cancers [28]. Marker *β-ACTIN* serves as a quality control. It was amplified by primers based on the same strategy as *EBNA1* gene. This marker could provide information if the bisulfite treatment is complete and if the template is of good quality. Recently, Hutajulu et al [18] investigated the methylation of *DAPK1*-promoter in NPC samples from Indonesia and The Netherlands. They also found some methylation of this promoter in healthy controls from the same area. One source of variation might depend on geographical and population-related differences in exposure to environmental factors. These differences may be reflected in different methylation patterns also in the control population. Ultimately one would of course like to identify promoters that can be discriminated based on methylation and can be used world-wide for screening. This aim can only be reached by studying many different populations. Hutajulu et al used different amplicon. We have screened 13 pairs of methylation primers located at different CpG sites of *DAPK1* promoter including primers published and the detection of methylation varied (data not shown). As methylation found in healthy subjects can be rather low, the variable results might also be affected by experimental conditions, sensitivity and cut-offs.

Taking EBV presence marker (*EBNA1*) plus tumor suggestive signal from at least one of the other three markers as a positive criteria, we achieved a diagnostic rate of 98% in 49 NPCs. Noticeably, we successfully detected all of eight T1 NPC patients included in this MMSP study, suggesting that the alternation of panel markers reflects changes on the early stage of nasopharyngeal carcinogenesis. It will pave the way to develop MMSP as a population-based screening tool. Pattern and timing of methylation status in certain genes are demonstrated to be associated with defined biological behaviors [29], thus MMSP assay will not only provide the diagnostic information, but also has the potential of predicting the specific behavior of individual tumors. Both EBV load and methylation level of certain gene have been shown to be capable of monitoring disease relapse after treatment, suggesting MMSP may serve as a way of long-term guardian during NPC treatment in the clinic. It may also be possible to extend the application of MMSP assay for the diagnosis of other types of cancer by evaluating MMSP pattern with different panel markers on tumor DNA from other body fluids, such as detecting prostate cancer or bladder cancer from urine, cervical cancer by cervical swab, etc.

To set up a functioning multiplex PCR reaction with five genes is challenging. All designed primers detecting different genes must be amplified optimally in the same reaction without competition or interference. Primers have to be chosen so that the PCR products can be separated well in gels. It is material saving, which is important when only small amounts of DNA are available from samples. It provides another advantage in that the semi-quantitative detection of methylated genes becomes comparable between the different genes tested. Once set up it is also time saving (one instead of five reactions and conditions).

Screening of high risk population with serologic methods, notably IgA anti-VCA has proven to be a very useful method to identify persons at high risk and early cases. Identification of persons with high IgA anti-VCA titers would be an important approach to identify the methylation pattern of promoters in early cases. Unfortunately at this stage we did not have access to such material from our study area. All our patients with suspicion of NPC have been tested for VCA-IgA by ELISA, before taking swab and biopsy. Almost all of the patients were EBV-VCA-IgA positive by ELISA. However in the patient records these ELISA results were only reported as positive or negative, without any indication of the titer level.

In future studies it would be highly relevant to combine EBV-serology with MMSP to test enhancement of sensitivity and specificity in order to find early cases and persons at high risk.

In summary, we developed a method which can simultaneously detect the presence of EBV DNA and assesses multiple gene methylation status using only picograms of tumor DNA from NP swabs. The method is easy to be handled in clinic and requires only routine equipments. We demonstrated the sensitivity and specificity of 98% and 100% respectively in detecting 49 NPC which include 19 early-stage patients. Further studies are required to testify the feasibility of MMSP assay as a population-based screening tool in NPC high-risk population as well as a way of monitoring tumor recurrence. Furthermore, the theoretical basis of MMSP may generate a new criterion for tumor classification from a molecular perspective. It will open the way to a host of innovative diagnostic, preventive and therapeutic strategies.
